# Dustiness of graphene and graphite

**DOI:** 10.1080/15459624.2026.2692355

**Published:** 2026-09-03

**Authors:** Douglas E. Evans, M. Christina Kander, Chen Wang, Peter B. Shaw, Seth McCormick, Kabir Rishi, Terrence S. Kuca, Derek E. Stinson, Gregory J. Deye, Matthew M. Dahm

**Affiliations:** a Chemical and Biological Monitoring Branch, Health Effects Laboratory Division, National Institute for Occupational Safety and Health (NIOSH), Centers for Disease Control and Prevention (CDC), Cincinnati, Ohio; b Emerging Technologies Branch, Division of Science Integration, National Institute for Occupational Safety and Health (NIOSH), Centers for Disease Control and Prevention (CDC), Cincinnati, Ohio; c Field Research Branch, Division of Field Studies and Engineering, National Institute for Occupational Safety and Health (NIOSH), Centers for Disease Control and Prevention (CDC), Cincinnati, Ohio

**Keywords:** Airborne dust, dust generation, exposure, inhalation, powder handling, powders

## Abstract

Graphene is among the most utilized nanoscale materials in the United States. It is a single-layer carbonaceous nanoscale material with a hexagonal structure. Subtractive synthesis from graphite is common in high-volume manufacturing, with graphene and graphite often present within the same workplace. There is little worker exposure data available to inform risk assessments, particularly for graphene. Dustiness determinations using EN15051–2 *Workplace Exposure—Measurement of the Dustiness of Bulk Materials. Part 2: Rotating Drum Method* were conducted for two graphene nanoplatelets, two graphene oxides, one few-layered graphene, one furnace carbon black, three natural and five synthetic graphites, and two coals. All 16 test materials were composed mostly of carbon. Dustiness determinations were compared to established thresholds for the inhalable, thoracic, and respirable particle size fractions. Measurements of specific surface area, moisture content, and unconsolidated bulk density were further obtained. Particles were imaged through scanning electron microscopy. The EN15051–2 test is a gentle gravity-induced procedure where the test material falls a limited distance. Dustiness determinations resulted in 2.2% or less of the test material collected in the inhalable size fraction, 0.89% or less in the thoracic, and 0.37% or less in the respirable subfraction. The thoracic fraction constituted ~31% of the inhalable fraction overall. The masses of four of five graphenes were below the gravimetric limit of detection of 0.57 μg in the respirable size fraction. Contributing factors were the gentle nature of the test and low unconsolidated bulk densities (5.9 to 174 kg/m^3^) for the nanoscale materials, resulting in 0.21 to 6.1 g loaded masses. Modest enhancements to the testing protocol could potentially improve quantification for low-density materials. All test materials and quantifiable size fractions were classified as moderate or high dustiness on a mg/kg basis. The carbonaceous materials in this study have a moderate to high propensity of becoming airborne when subjected to a gentle gravity-induced stimulus. Without adequate controls to prevent dust emissions, handling could result in inhalation, dermal, and mucous membrane exposures as well as present slip, electrical, fire, and dust explosion hazards.

## Introduction

Graphene is one of the most utilized nanoscale materials in the United States (Lee et al. 2016; Babik et al. 2018; Mbayachi et al. 2021; McCormick et al. 2021). Nanomaterials, nano-objects and nanoplates have at least one external dimension less than 100 nm (ISO 2023). Graphene is a two-dimensional material where carbon atoms are tightly bound in a hexagonal structure (Geim and Novoselov 2007; ISO 2020). Commercial graphenes can be single-layer, bilayer, or few-layer (3 to 10) structures (Xu et al. 2010; Zhou et al. 2018). Electrical properties (band overlap) become indistinguishable from graphite at 11 or more carbon layers (Partoens and Peeters 2006). Derivatives include oxides, reduced oxides, nanoplatelets, nanoribbons, and graphene quantum dots (Nguyen and Nguyen 2016; Tiwari et al. 2020). Properties include high strength, low density, high electrical and thermal conductivity, flexibility and transparency (Katsnelson 2007; Lee et al. 2008; Ferrari et al. 2015; Gwalani et al. 2024), with applications in the electronics, medical, energy storage, composites, and water treatment industries (Future Markets 2019).

Graphite is a three-dimensional crystalline allotrope of carbon comprising many graphene layers. It is an important precursor to graphene production and is consumed on a large scale. Graphite occurs naturally or is synthesized. Natural (amorphous, flake, crystalline lump or vein) graphite is mined, while synthetic graphite is derived from carbonaceous materials at high temperatures. Applications include uses in medicine, thermal management, recarburising in steel and other alloy manufacturing, composites, batteries, castings, refractories, electrodes, and lubricants (ECGA, 2026).

There are multiple approaches to graphene synthesis (Novoselov et al. 2005; Nguyen and Nguyen 2016; Tiwari et al. 2020; Mbayachi et al. 2021). Mechanical exfoliation is a subtractive (top-down) synthesis where graphene flakes are mechanically sheared from graphite (Shams et al. 2015; Bhuyan et al. 2016; Papageorgiou et al. 2017; Kuziel et al. 2020; Sumdani et al. 2021). Chemical exfoliation is a two-step, subtractive process. Intercalating molecules increase interlayer spacing (Bhuyan et al. 2016). Sonication or ultracentrifugation follows, exfoliating graphene (Green and Hersam 2009, 2010; Park et al. 2009; Marcano et al. 2010). Chemical vapor deposition, a gaseous additive approach (Shams et al. 2015; Saeed et al. 2020; Mbayachi et al. 2021; Sun et al. 2021; Choi et al. 2022), is utilized in pristine graphene production (Ferrari et al. 2015; Bhuyan et al. 2016; Lee et al. 2016). Subtractive synthesis is favored for high-volume production.

Occupational exposures to graphene are of concern (Guo and Mei 2014; Shams et al. 2015; Pelin et al. 2018; McCormick et al. 2021). Graphene accumulates in several tissues and organs, potentially resulting in lung injury, chronic obstructive pulmonary disease, fibrosis, and extrapulmonary effects (Ema et al. 2017; Kim et al. 2018; Pelin et al. 2018; Xiaoli et al. 2020; Poulsen et al. 2021; Niang et al. 2025). Graphene toxicity may ultimately depend on specific material type, hydrophobicity, surface chemistry, size, dosage, and duration of exposure, in addition to other factors (Kong et al. 2024).

Target organs associated with graphite exposure include the respiratory and cardiovascular systems. Adverse health outcomes include dyspnea (difficulty breathing), black sputum, decreased pulmonary function, lung fibrosis, and cough (NIOSH 2020; Cohen et al. 2023).

Occupational routes of exposure include inhalation, dermal, ocular, and ingestion, with inhalation of primary concern. During manufacturing, workers can be exposed to graphene or graphite as dust emissions (Brouwer et al. 2006; O’Shaughnessy et al. 2012; Evans et al. 2013). Operations that generate airborne dust include powder loading, mixing, pouring, packing, transport, falling powders as a transfer, spill, cleanup, equipment maintenance, or air movement over powders (Hammond 1980; Cowherd 1981; Dennis and Bubenick 1983; Heitbrink et al. 1992; Boundy et al. 2006; Lidén 2006; Evans et al. 2010; Dahm et al. 2012; Evans et al. 2013; NIOSH 2024).

Several occupational exposure limits (OELs) have been promulgated in the United States for carbonaceous materials (Table 1). Carbonaceous OELs span four orders of magnitude (10,000×) in airborne concentrations. Materials include synthetic and natural graphite, carbon black, lignite, bituminous, and anthracite coals, carbon nanotubes, and carbon nanofibers (NIOSH 2013, 2020; ACGIH 2026). No OELs yet exist for graphene (Spinazzè et al. 2016). However, occupational hazard/exposure banding has suggested that some graphene materials may elicit adverse health effects at relatively low exposure concentrations, i.e., ≤0.01 mg/m^3^ (Niang et al. 2025).

### Approach

With little exposure data (Poikkimäki et al. 2025; Dahm et al. 2026) and increased graphite and graphene production, dustiness determinations can help fill critical exposure and risk assessment knowledge gaps by providing a relative measure of inhalation exposure potential and the particle size fractions likely encountered. Dustiness may be defined as the propensity of a finely divided solid to form an airborne dust (aerosol) by a mechanical or aerodynamic stimulus (NIOSH 2024). Dust emissions and resulting airborne concentrations depend on many factors, including quantities of materials, the process, ventilation (dilution), and the presence of engineering controls (Fransman et al. 2011; Ribalta et al. 2019). Historically, dustiness has been strongly correlated with inhalation exposure (Heitbrink et al. 1989; Brouwer et al. 2006; Ribalta et al. 2019). Testing should replicate dust generation mechanisms in workplaces as closely as possible (Evans et al. 2013). Professionals should select a method closely simulating processes responsible for exposures (CEN 2013a).

There are many dustiness test methods. Some have been further standardized (NIOSH 2024). Arguably the most developed and widely adopted is the EN15051–2 *Workplace Exposure—Measurement of the Dustiness of Bulk Materials. Part 2: Rotating Drum Method* (CEN 2013b, NIOSH 2024). Originally developed for industrial minerals (Lyons and Mark 1994; HSE 1996; Lyons et al. 1996), the method has since been utilized in multiple applications (Burdett et al. 2000; CEN 2006; Tsai et al. 2009; HSE 2010; Burdett et al. 2013; Mason et al. 2017; CEN 2019; Chen et al. 2021). The test effectively simulates many industrial processes, transfer operations, rotational mixing, and conveyance, where materials may fall a limited distance. The resulting airborne dust is evaluated from an inhalation exposure perspective. It is a gentle test with respect to the energy imparted to the test material. The test material falls repeatedly under the influence of gravity, no more than 300 mm in height over a period of 1 min, and the air flows are laminar (Chen et al. 2021). As the substrate angle periodically increases, the test powder exceeds its angle of repose, and a local avalanche ensues, aerosolizing a fraction of the falling test powder. The gentle horizontal air flow transports the airborne dust into the sampling section, where the entry geometry and two porous size-selective foams and a filter in series provide the inhalable (d_50_ = 100 μm), thoracic (d_50_ = 10 μm), and respirable (d_50_ = 4 μm) particle size fractions simultaneously (CEN 1993; ISO 1995; Vincent et al. 1999). The inhalable size fraction describes the likelihood of particles entering the human nose or mouth. The thoracic fraction describes the likelihood of particle penetration past the larynx and into the conducting airways. The respirable size fraction describes the probability of particles of a given size penetrating the terminal gas exchange regions of the airways (alveoli). The thoracic and respirable fractions are defined as subfractions of the inhalable fraction. The inhalable, thoracic, and respirable penetration curves are provided in NIOSH (2024).

The dustiness of 16 carbonaceous test powders was investigated, comprising mostly graphene and graphite materials. The materials were further characterized by the physical attributes of specific surface area, moisture content, unconsolidated (untapped) bulk density, and particles imaged by scanning electron microscopy. Graphene is commonly derived from graphite in subtractive synthesis; therefore, graphite and graphene may be present within the same workplace. To compare with other common carbonaceous materials, a furnace carbon black and two coals were included in this study.

## Methods

*Dustiness determinations* were conducted with an EN15051–2 (CEN 2013b) rotating drum (JS Holdings, Stevenage, UK). Measurements were mass-based, with collected airborne dust (mg) compared to the loaded test material (kg) for inhalable, thoracic, and respirable respiratory size fractions (CEN 2013b; NIOSH 2024).

The EN15051–2 drum consisted of an electrically grounded stainless-steel cylinder (300 mm diameter) with conical entry and exit (Figure 1). Eight internal longitudinal vanes (230 mm length x 25 mm height) protruded radially. The vanes lifted and dropped the test powder repeatedly as the drum rotated for 1 min. Air was drawn through the apparatus, through a radial rotating coupling and flexible tubing at 38 L/min with a vacuum pump. Flow was set with a flowmeter (4000 Series, TSI Instruments Ltd, Shoreview, MN) positioned between the apparatus and the vacuum pump.

Conditioned air, 21 ± 2 °C and 50 ± 2% RH, at 40 L/min (HCS-501, Assay Technology, Inc., Livermore, CA, USA) was supplied to the entry through a conical inlet and rotating union (Figure 1). Excess conditioned air (~2 L/min) was vented to the atmosphere through a tee-fitting (Figure 1, top). Temperature and relative humidity were assessed (HUMICAP HM70, Vaisala, Vantaa, Finland). The conical filtered inlet (Figure 1, bottom left) and conditioned air supply were carefully considered, and necessary deviations to EN15051–2, which otherwise permits monitored ambient laboratory air to be drawn into the apparatus (CEN 2013b). Seasonal indoor humidity variations ranging from ~75% RH annually (Cincinnati, USA) can result in an uncontrolled variable inconsistently influencing testing (NIOSH 2024).

Conditioned air passed through a 150 mm borosilicate glass microfiber filter (Grade GF/A Whatman, Maidstone, UK), which diffused the incoming air, prevented the test powder from escaping, and prevented any contaminants from entering the apparatus. The laminar flow entered the central dust generation section, transporting freshly generated airborne dust to the conical exit and the sampling section.

Within the sampling section, dust was collected by two nickel particle-size-selective foams of 80 mm diameter (JS Holdings, Stevenage, UK) and a borosilicate glass microfiber filter of 81 mm diameter (Grade GF/A, Whatman, Maidstone, UK). Foams were 20 and 80 ppi (pores per inch) and 20 and 12 mm thick (CEN 2013b; NIOSH 2024). The serial sampling system provided the inhalable, thoracic, and respirable particle size fractions by aerodynamic diameter (CEN 1993; ISO 1995; Vincent et al. 1999). The inhalable fraction consisted of material collected on both porous foams and a filter. The thoracic fraction consisted of material collected on the second foam and filter. The respirable fraction consisted of airborne material passing through both foams and collected solely on the filter.

Two graphene nanoplatelets, two (low O content) graphene oxides, one few-layered graphene, one furnace carbon black, three natural graphites, five synthetic graphites, and two coals were studied. Materials were selected based on industrial relevance or high-volume commercial production. Product codes and manufacturing approach are presented (Table 2). Supplementary materials provide test material provenance.

Representative samples were obtained by inverting containers several times before extraction in accordance with EN15051–1 Workplace Exposure—Measurement of the Dustiness of Bulk Materials. Part 1: Requirements and Choice of Test Methods (CEN 2013a). Materials were conditioned in thin layers at 21 ± 2 °C and 50 ± 2% RH within a chamber for 48 h in accordance with more stringent conditioning in EN17199–2 *Workplace Exposure—Measurement of Dustiness of Bulk Materials that Contain or Release Respirable NOAA and Other Respirable Particles. Part 2: Rotating Drum Method* (CEN 2019). Conditioned air was supplied to the chamber (HCS-501, Assay Technology, Inc., Livermore, CA, USA).

Material transfers were conducted within a laminar-flow powder handling hood (XPert Nano Enclosure, Labconco, Kansas City, MO, USA). Dustiness determinations utilized 35 mL of conditioned test material. Stainless steel 35 mL vials were filled, without tapping or pressing (unconsolidated), until heaped, leveled using a straight spatula handle, and weighed.

A stand consisting of 50 mm PVC elbows and pipe stabilized the cylindrical drum during assembly, loading and disassembly. The sampling system, consisting of two foams and a filter, was assembled with annular static-dissipative PTFE gaskets to reduce dust transfer between media (Figure 2). Test powder was added evenly along the full length within the drum base. Vials were post-weighed. The inlet was assembled, the drum placed on the roller assembly, and coupled to the conditioned air and vacuum (Figure 1). Dustiness determinations proceeded for 1 min at four revolutions per minute (RPM), with five replicate determinations for each test material.

The drum was uncoupled and placed within the hood for safe disassembly. The sampling section was carefully disassembled, and foams and filter placed in clean petri dishes for post-conditioning and weighing. The inlet was disassembled. The drum was vertically aligned above a wide-mouth beaker, lined with a sealable bag. External tapping with a rubber mallet removed loose residual test material. Collection and reuse of test material may be warranted where materials are cost-prohibitive or in limited supply. Thorough cleaning occurred between different test materials. Residues were vacuumed (Nilfisk, GMPJ115, Brooklyn Park, MN, USA) followed by an alcohol wipe down. Sampling components, including reusable nickel foams, were consistently washed (Steamscrubber 44003 series, Labconco, Kansas City, MO, USA) with ~20 mL of low-foaming liquid detergent. Cycles consisted of prewash, wash, two tap-water rinses, three deionized water (DW) rinses, and drying, all at 60 °C. DW rinses removed soluble mineral residues. Foams were dried at 120 °C (Isotemp 180 L Oven FA, Thermo-Fisher Scientific, Waltham, MA, USA).

Gravimetric weighing of filters, pre- and post-determinations, was conducted with a seven-place analytical balance (XPR6U Ultra-Micro Comparator with filter option #30300922, Mettler Toledo, Columbus, OH, USA). The draft shield aperture was custom enlarged to accommodate oversize (> 70 mm) filters. Filters were conditioned at 21 ± 2 °C and 50 ± 2% RH within a controlled chamber overnight and passed through an antistatic ionizer (PRX U, Haug, Williamsville, NY, USA) multiple times before weighing. A five-place analytical balance (XPE105, Mettler Toledo, Columbus, OH, USA) was utilized for the foams and vials. Foams were passed through an antistatic ionizer and weighed immediately pre- and post-determinations. Three replicate measurements were conducted for each filter and foam. Sample media were stored in glass Petri dishes. To minimize material loss or contamination, foams and vials were handled at the edges with clean gloved hands, and filters at the edges with tweezers. Gravimetric weighing was guided by ISO (2009).

Specific surface areas (m^2^/g) were determined with nitrogen adsorption BET analyses (Brunauer et al. 1938; ISO 2022) with a TriStar II 3020 surface area and porosity measurement system (Micromeritics Instrument Corp., Norcross, GA). Two grams of each test material were required. Samples were pre-conditioned in nitrogen at 300 °C for 4 hr. Ultra-high purity nitrogen gas within a liquid nitrogen bath was utilized for analyses. For graphene, carbon black, and graphite materials, eight-point determinations were performed, and for coals, five-point determinations were performed. Coals required extended instrument run time due to restricted pore structure. Three replicates were performed.

Moisture content (%) for each test material was determined through oven-drying (CEN 2013a). Clean glass Petri dishes were initially weighed. Two grams of test material were added. Materials were conditioned to 50 ± 2% relative humidity and 21 ± 2 °C for several hours. A further gravimetric measurement was taken. Test materials were heated to 120 °C overnight. Samples were cooled within a desiccator, followed by a final gravimetric measurement. The mass loss through oven-drying was compared to the conditioned mass at 50% RH. Three replicates were performed.

Unconsolidated bulk density (kg/m^3^) was determined by loading the 35 mL vial with test material, and the mass loaded into the drum apparatus was determined gravimetrically. Each dustiness determination provided a bulk density measurement (NIOSH 2024).

The mean and one standard deviation of replicates were obtained for dustiness in the inhalable, thoracic, and respirable size fractions (mg/kg), specific surface area (m^2^/g), moisture content (%), and bulk density (kg/m^3^).

Test materials were dispersed in isopropanol (0.1 mg/mL). Following sonication and agitation, 0.1–0.2 mL was applied to gold-coated glass slides and dried. Slides were analyzed using a Phenom XL scanning electron microscope (SEM) with secondary electron (SE) detection in high-vacuum mode (~1 Pa) at 15 kV and 1.7 nA.

## Results

### Gravimetric limits of detection

With a clean test apparatus, 10 replicate dustiness determinations without test material were conducted to estimate error associated with all aspects of transfer, storage, conditioning, and gravimetric weighing of clean collection media. Conditioned filters (~281 mg) resulted in a gravimetric limit of detection (three standard deviations) of 0.57 μg through pre- and post-gravimetric weighing. Similarly, 20 ppi foams (~49 g), 80 ppi foams (~43 g), and 35 mL vials (~52 g) resulted in gravimetric limits of detection of 97, 85, and 1.4 *μ*g.

### Dustiness determinations

Sixteen preconditioned test materials were characterized with the EN5051–2 rotating drum. Determinations were compared to four classification categories, very low, low, moderate, and high dustiness for the inhalable, thoracic, and respirable particle size fractions (Table 3; CEN 2013b). Classification thresholds were established from the quartiles of over 220 historical test materials (CEN 2013b). Mean averages (mg/kg) and one standard deviation of five replicates for each test material are provided (Table 4).

Respirable masses (collected solely with filters) for two nanoplatelets (R-25 and C-500), few-layered graphene (F030), and edge-oxidized graphene (EOG) fell below the gravimetric limit of detection and are not reported. For calculating the thoracic and inhalable fractions, a value of LOD/√2 was substituted for these respirable values (NIOSH 2016a).

Inhalable values ranged from 1,035 (synthetic graphite, M_304_) to 21,781 mg/kg (natural graphite, E_110_), a range of approximately 21 times. Thoracic values ranged from 395 (edge-oxidized graphene, EOG) to 8,947 mg/kg (carbon black, Printex 90), a range of approximately 23 times. Quantifiable respirable values ranged from 103 (synthetic graphite, M_304_) to 3,689 mg/kg (graphene oxide, B050), a range of approximately 36 times. Graphene oxide B050 (3,689 mg/kg) had the highest respirable value, which was approximately 11 times greater than synthetic graphite 4,147 (331 mg/kg), the next highest respirable value. Comparing quantifiable dustiness results to EN15051–2 dustiness classifications, all values (mg/kg) were moderate or high (Tables 3 and 4).

### Comparing size fractions

Figure 3 is a scatterplot of mean thoracic and inhalable dustiness (mg/kg) for all 16 carbonaceous test materials, derived from values in Table 4. Materials were further grouped by type: graphenes, graphites, coals, and carbon black. The thoracic fraction is a subfraction of the inhalable. A good linear correlation (R^2^ = 0.96) was obtained between inhalable and thoracic dustiness for all 16 carbonaceous materials overall. This linear relationship took the form D_Thor_ = m*D_Inh_ + b, where slope *m* = 0.31 (95% confidence intervals of 0.27 and 0.35) and intercept *b* = 1.85 (95% confidence intervals of −2.23 and 5.83). The thoracic fraction contributed ~31% of the inhalable fraction overall for all carbonaceous materials in this study. This was the only significant relationship observed between measured variables. More scatter was evident for the nanoscale materials, and a closer relationship was evident with microscale graphites and coals. In a prior study, the thoracic fraction comprised ~26% of the inhalable fraction for nanoscale calcium carbonates (Burdett et al. 2013).

Figure 4 is a scatterplot associating mean respirable and inhalable dustiness (mg/kg) for 12 test materials, from values in Table 4. The respirable fraction is a subfraction of the inhalable. For 10 graphites and coals, respirable dustiness contributed ~4% to the inhalable. In a prior study, respirable dustiness constituted 3% of the inhalable fraction for nanoscale calcium carbonates (Burdett et al. 2013). B050 single layer oxide, the only graphene material quantified, and Printex 90 carbon black provided more scatter. Respirable dustiness contributed 82% to inhalable dustiness for B050 single-layer graphene oxide, and 0.8% for Printex 90 carbon black, a 102× difference in their contributions. The B050 single-layer oxide possessed a low unconsolidated bulk density of 5.9 kg/m^3^ (Table 4). In prior work, HiPCO single-walled carbon nanotubes, a low-density material, had a respirable fraction constituting 84% of total dust (Evans et al. 2013). Again, in prior work, the respirable fraction constituted ~39% of the total dust overall, for twenty-seven fine and nanoscale materials, dispersed within an energetic aerodynamic dustiness test apparatus (Evans et al. 2013).

Figure 5 is a scatterplot of mean respirable and thoracic dustiness for 12 materials, derived from values in Table 4. The respirable fraction is a subfraction of the thoracic fraction. Scatter is evident with B050 single-layer graphene oxide and Printex 90 carbon black, the two nanoscale materials. Respirable dustiness constituted ~87% of thoracic dustiness for B050 and ~1.5% for Printex 90. The respirable and thoracic dustiness relationship was more consistent for the 10 microscale graphites and coals and contributed ~11% to thoracic dustiness overall (4 to 20% range). In a prior study, respirable dustiness constituted ~12% of thoracic for nanoscale calcium carbonates (Burdett et al. 2013).

### Physical properties

BET Specific surface areas (SSAs) ranged from 0.9 (coal PPC 1157) to 595 m^2^/g (graphene nanoplatelets C-500), a range of 660 times the lowest value overall. Graphenes ranged from 18 (F030) to 595 m^2^/g (C-500) and possessed the highest SSAs overall. Carbon black (Printex 90) had an SSA of 273 m^2^/g. Graphite materials ranged from 2.0 (K_118_) to 351 m^2^/g (4,147). Coals possessed the lowest SSAs overall at 0.9 (PPC 1157) and 2.9 m^2^/g (BHF).

Moisture contents ranged from 0.014 (L_102,_ synthetic graphite) to 5.6% (B050, single layer graphene oxide), a range spanning 400x of the lowest value overall. Graphene ranged from 0.11 (R-25 nanoplatelets) to 5.6% (B050). Carbon black (Printex 90) had 2.5%. Natural graphite ranged from 0.057 (E_110_) to 0.13% (M_100_). Synthetic graphite ranged from 0.014 (L_102_) to 3.7% (4,147). With the notable exception of synthetic graphite 4147 (3.7%), moisture content for graphites did not exceed 0.2%. Coals (PPC 1157 and BHF) possessed moisture contents of 1.3%. Graphite possessed the lowest, and graphene the highest moisture contents overall. Greater moisture content was associated with greater SSA, but with significant scatter evident. Moisture can significantly influence dustiness through capillary interactions, although the relationship is complex (NIOSH 2024).

Unconsolidated bulk density ranged from 5.9 (single-layer graphene oxide, B050) to 676 kg/m^3^ (synthetic graphite, K_118_), a range spanning 115 times the lowest value overall. For graphene, densities ranged from 5.9 (B050) to 174 kg/m^3^ (EOG). Carbon black (Printex 90) possessed a density of 135 kg/m^3^, falling within the graphene (nanoscale) density range. Graphite ranged from 252 (F_117_) to 676 kg/m^3^ (K_118_). Coals possessed densities of 396 (BHF) and 592 kg/m^3^ (PPC 1157), falling within the graphite (microscale) range. Microscale materials possessed the greatest bulk densities, and nanoscale materials the least. See Figure S1. The physical properties of specific surface area, moisture content, and unconsolidated bulk density were not reliable predictors for dust-generating behavior.

Electron microscopy provided the images presented in Figure 6 for four nanoscale materials. Graphene nanoplatelets R-25, few-layered graphene F030, and single-layer graphene oxide B050 exhibited folded-sheet structures. Furnace carbon black, Printex 90, exhibited more compact particles with grape-like fractal aggregates present. Figure 7 depicts two microscale graphite materials, natural graphite E_110_ and synthetic graphite L_102_. Both exhibited compact morphologies. Compact morphologies align with greater bulk densities for microscale materials overall (Table 4).

## Discussion

### EN15051–2 dustiness determinations

Dustiness determinations provide EHS professionals and product manufacturers with an improved understanding of the dust emission potential that may result in the workplace. The EN15051–2 rotating drum is a relatively gentle test from the perspective of the potential energy applied to the test material. Airborne dust that is ultimately collected is a small fraction of the initial test material overall. The test powder falls multiple times from a limited (less than 300 mm) height under the influence of gravity for 1 min. Our dustiest materials overall, E_110_ natural graphite (21,781 mg/kg) and Printex 90 carbon black (17,955 mg/kg), resulted in ~2.2 and ~1.8% of the loaded test material collected as airborne dust in the inhalable fraction. In a prior study, the dustiest nanoscale calcium carbonate (13,845 mg/kg) resulted in ~1.4% as airborne dust in the inhalable fraction (Burdett et al. 2013). The moderate-to-high dustiness threshold is 3,000 mg/kg (0.3%) in the inhalable size fraction.

The least dusty materials overall, synthetic graphite M_304_ (1,035 mg/kg) and K_118_ (1,358 mg/kg), resulted in 0.10 and 0.14% of the test material collected as airborne dust in the inhalable size fraction (moderate dustiness). M_304_ had the smallest and K_118_ the largest bulk particle size distribution for synthetic graphites from the same manufacturer (Table S2). Four of five graphenes (R-25, C-500, F030, B050) fell into high dustiness categories for the inhalable and thoracic size fractions. The edge oxidized graphene (EOG) is in the high dustiness category for inhalable, and the moderate dustiness category for the thoracic size fraction. Single layer graphene oxide B050, natural graphite E_110_, synthetic graphites 4147 and 240, and coal PPC 1157 were within the high dustiness category for each size fraction.

Only airborne particles that escaped the drum were collected and quantified (NIOSH 2024). The sampling section has experimentally determined particle penetration characteristics closely matching the inhalable penetration efficiency curve (Burdett et al. 2000). Numerical modeling has suggested optimal particle sizes (~8 to ~15 μm) preferentially migrating from the outer drum walls, being transported by the airflow, and being subsequently collected (Chen et al. 2021).

Good reproducibility was observed for all 16 test materials. The relative standard deviation (RSD, one standard deviation divided by the mean, as %) for the inhalable fraction ranged from 4 to 22%. Synthetic graphite 240 and few-layered graphene F030 were very consistent (4%). For the thoracic fraction, RSDs ranged from 5 (R-25 graphene nanoplatelets) to 30%. RSDs for the quantifiable respirable fraction ranged from 3 to 17%. Printex 90 carbon black (3%), synthetic graphite 4147 (3%), and synthetic graphite 240 (4%) were very consistent. High precision (low RSDs) for many materials and low gravimetric limits of detection suggested that laboratory procedures were remarkably consistent. Greater RSDs (up to ~30%) suggested variation between replicate determinations in airborne dust generation for certain test materials.

### Implications for the workplace

Aerosol sampling for the inhalable fraction is common, whereas thoracic sampling is less frequent. When absent from exposure assessment, an estimate for the thoracic fraction can be deduced from inhalable data. The thoracic fraction constituted ~31% of the inhalable fraction overall for all carbonaceous test materials in this study, based on the linear regression. For the six nanoscale materials (graphenes and carbon black), the thoracic fraction constituted ~56%, and for the 10 microscale materials (graphites and coals), the thoracic fraction contributed ~38% of the inhalable fraction. In a prior study, the thoracic fraction contributed ~26% of the inhalable fraction for nanoscale calcium carbonates (Burdett et al. 2013).

All materials and quantifiable size fractions fell into moderate or high dustiness categories. Dust generation is relatively easy for these carbonaceous test materials. Graphites and graphenes are utilized for their bulk lubricating properties, derived from relatively weak interparticle forces. Enhanced airborne dust generation leads to increased inhalation and safety risks if not adequately controlled (Risk = Exposure × Hazard). Enhanced dustiness may require higher-performing exposure or safety controls in the workplace. The hierarchy of controls is the preferred order in which controls may be considered (NIOSH 2023). Reduced dust products and processes, perhaps developed through dustiness testing, can play an important role in prevention through design, as potential substitution controls (ANSI/ASSP 2021).

Carbonaceous materials potentially pose fire, flash fire, and significant dust explosion hazards. With K_st_ values (the maximum rate of pressure rise) of up to 200 barm/s and p_max_ (the maximum overpressure) potentially exceeding 7 bar, these materials generally fall within explosion class St-1 (Turkevich et al. 2015, 2016). Carbonaceous materials are also electrical conductors. As airborne dust, conducting materials may lead to electrical short-circuiting, potentially damaging equipment, electrical infrastructure, and/or becoming a source of ignition (OSHA 2005; NFPA 2022). Areas or zones with combustible carbon dust present require a Class II designation (combustible dust hazards). Division I (high risk) pertains to sufficient dust quantities present to produce an explosion under normal operating conditions, and Division II (low risk) pertains to dust quantities that do not normally exist but potentially could through equipment or other failures. Although typically falling within Group F (carbonaceous dusts), conductive materials could potentially require more stringent mitigation requirements typically reserved for Group E (metal dusts) (NFPA 2024). Electrical equipment requirements may include dust-tight, explosion-proof, bonding/grounding, and/or limited surface temperatures, for example (NFPA 2024, 2025, 2026). In addition, the relative ease with which these materials become airborne (high dustiness) could potentially increase the likelihood of crossing minimum explosible concentration (MEC) thresholds.

Lubricants or glidants such as graphene and graphite, in some applications, present in small quantities, can reduce friction and result in slips. Economic considerations resulting from uncontrolled dusts may include product loss, potential for cross-contamination, degradation in product quality, cleanup (housekeeping), and premature equipment failure (NIOSH 2024).

### Limitations

Two graphene nanoplatelets, two (low O) graphene oxides, and one few-layered graphene were studied. Studying additional graphene materials could have provided further insights into dust-generating behavior.

Laboratory procedures provided high precision with limits of detection of 0.57 (filters), 85 and 97 μg (nickel foams), and 1.4 μg (vials). Four graphenes were not quantifiable for the respirable fraction but exhibited moderate or high dustiness in the thoracic and inhalable fractions. The quantifiable graphene (B050) exhibited high dustiness in the respirable fraction.

Contributing factors to very small respirable masses (filters) were twofold. First, EN15051–2 is a relatively gentle dust-generating procedure. 2.2% or less of loaded test materials were collected in the inhalable fraction, 0.89% or less in the thoracic, and 0.37% or less in the respirable subfraction. Second, there is the small (35 mL) test volume requirement. Unconsolidated bulk densities for six nanoscale materials (Table 4) were 5.9 to 174 kg/m^3^. Consequently, loaded masses were only 0.21 to 6.1 g (Table S3). In contrast, with greater unconsolidated bulk densities (252 to 676 kg/m^3^) for 10 microscale test materials, loaded masses were 8.8 to 24 g. Loaded test material masses ranged approximately two orders of magnitude (100x) overall.

For all test materials, less than 5.0 μg of airborne dust was collected on filters, constituting the respirable fraction. Seven test materials resulted in ~1.0 μg or less. Quantifying the respirable fraction for many materials in this study was possible only with the precision afforded by a seven-place analytical balance. EN15051–2 (CEN 2013b) stipulates a minimum five-place analytical balance (10 *μ*g readability), with the limit of detection ultimately influenced by collection media.

A density threshold of ~200 kg/m^3^ (or ~7 g loading) was observed. Test materials with lower unconsolidated densities were more challenging to quantify in the respirable fraction (Figure S1). Enhanced testing protocols may be warranted in these instances. High-precision analytical balances, extended run time, increased test material loading, or combinations of these enhancements may be viable approaches for quantifying low-density test materials.

The EN15051–2 rotating drum is arguably the most developed and widely adopted dustiness test system worldwide for assessing workplace inhalation hazards (NIOSH 2024). It is an excellent tool for simulating low to moderate energy dust dispersion mechanisms, those routinely encountered within the workplace, i.e., materials fall a limited distance. Although perhaps less frequent, there may be instances where high-energy dust dispersion occurs. The EN15051–2 rotating drum may underestimate the potential for dust dispersion in these instances and the size fractions that result. A complementary aerodynamic test system, for example, may be better suited for simulating high-energy dust dispersion scenarios (NIOSH 2024).

Low-density nanoscale materials may potentially provide challenges for air sampling in the workplace. Since OELs are predominantly mass-based, larger air volumes may be required for collecting sufficient quantifiable mass for subsequent gravimetric or chemical analyses. Greater sampling flow rates, longer sampling durations, and smaller filter areas (elemental carbon analysis, NIOSH 2016b) may improve detection.

## Conclusions

EN15051–2 is a gentle gravity-induced procedure, with test materials falling a limited (less than 300 mm) distance. Dustiness determinations resulted in ~2.2% or less of the loaded test materials collected in the inhalable fraction, ~0.89% or less in the thoracic, and ~0.37% or less in the respirable subfractions. Four graphenes in the respirable size fraction were below the gravimetric limit of detection of 0.57 μg. The relatively gentle test, coupled with low unconsolidated bulk densities for these materials were contributing factors.

A five-place analytical balance, the minimum requirement stipulated by EN15051–2, is arguably insufficient for quantifying the respirable dustiness of low-density materials. Improved quantification can be achieved with high-resolution balances. Further enhancements might include extended run time, increased test material loading, or combinations of the above.

All test materials and quantifiable size fractions were classified as either moderate or high dustiness on a mg/kg basis. A positive correlation was observed between thoracic and inhalable dustiness for all carbonaceous test materials. The thoracic fraction constituted ~31% of the inhalable fraction overall. No other significant correlations were observed.

The carbonaceous materials in this study have a moderate to high propensity of becoming airborne when subjected to a gentle gravity-induced stimulus. Falling materials are common in the workplace. Handling, conveying, and processing of these materials, without adequate controls, could result in inhalation, dermal, and mucous membrane exposures as well as present slip, electrical, fire, and dust explosion hazards.

## Supplementary Material

Supplemental

Supplemental data for this article can be accessed online at https://doi.org/10.1080/15459624.2026.2692355. AIHA and ACGIH members may also access supplementary material at http://oeh.tandfonline.com.

## Figures and Tables

**Figure 1.**
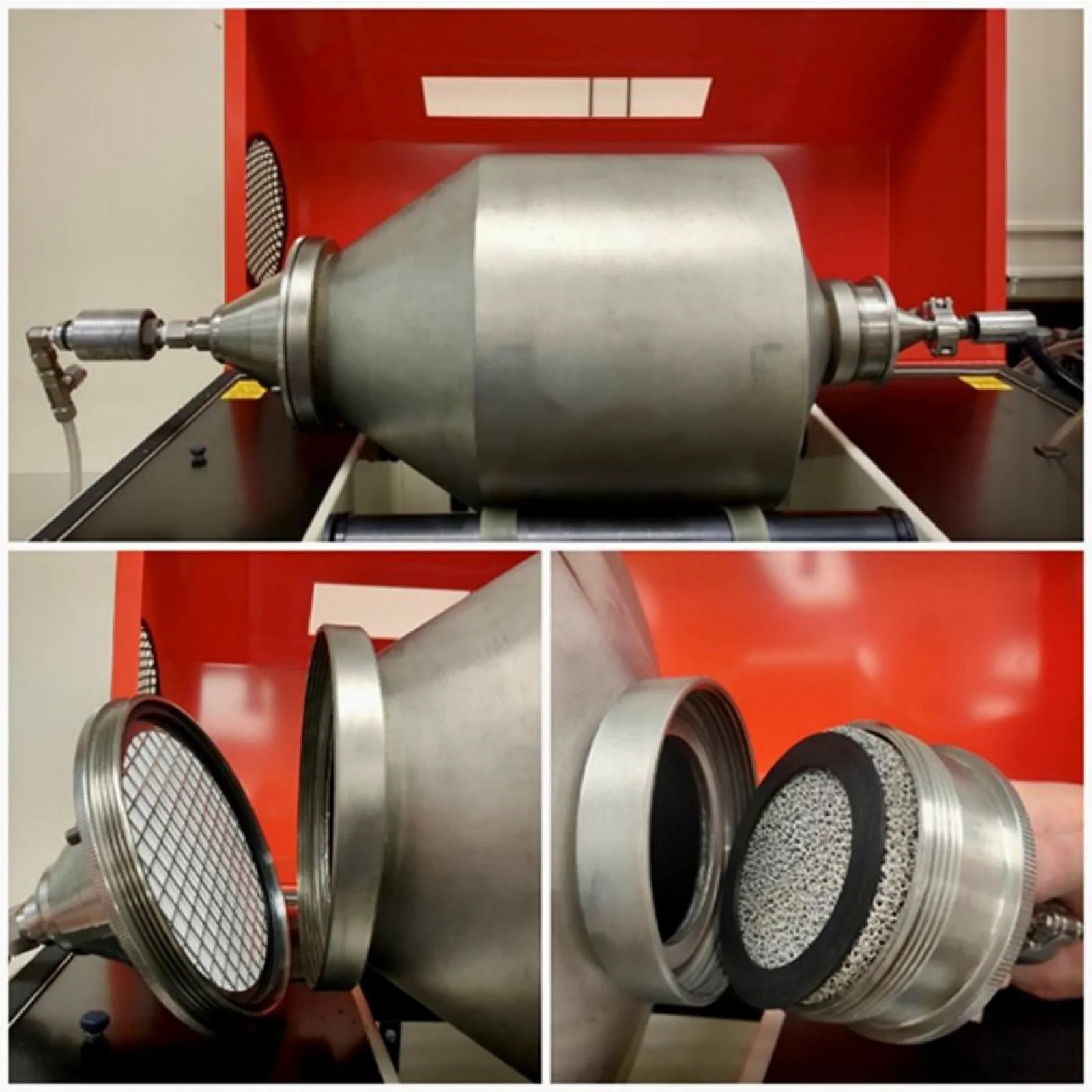
The assembled rotating drum (top) situated on the rollers and chassis. The conical filtered inlet (bottom left) and complete sampling assembly (bottom right) are shown uncoupled from the drum. Air moves from left to right. Eight internal longitudinal vanes (not shown) protrude radially.

**Figure 2.**
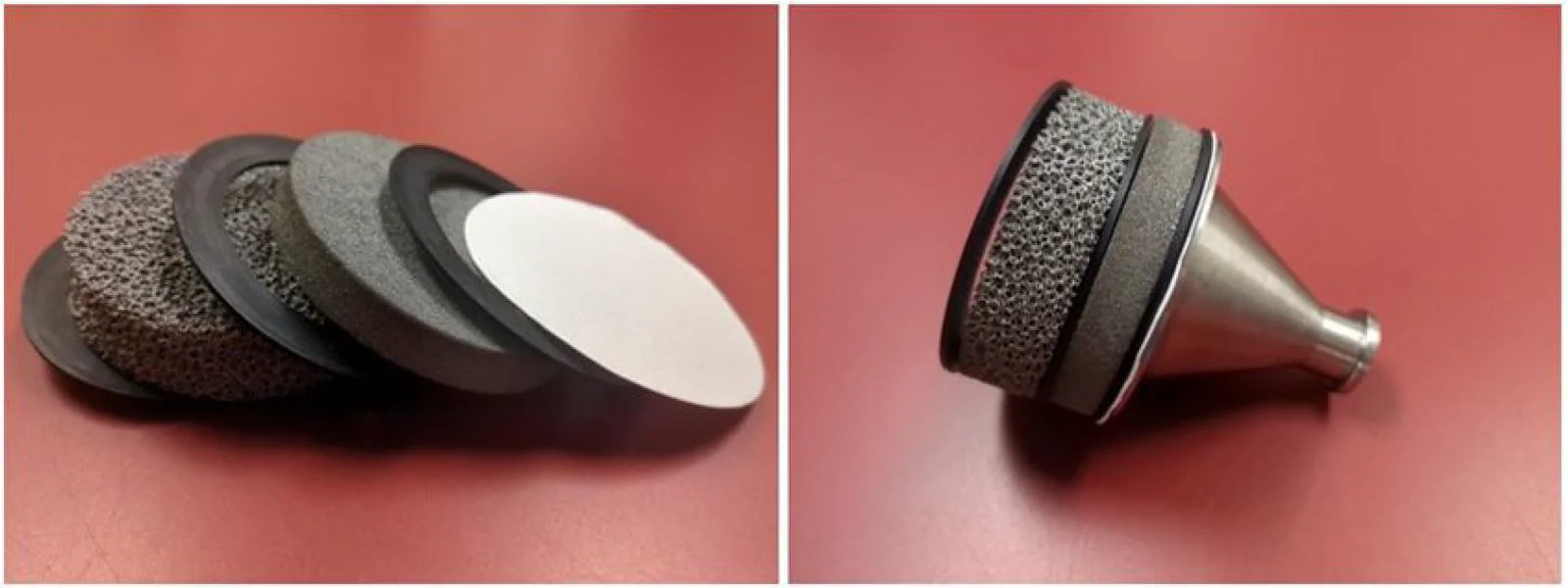
Sampling collection media. From left to right: 20 ppi nickel foam, 80 ppi nickel foam, borosilicate glass fiber filter with black, annular, static-dissipative PTFE gaskets (2 mm thick) sandwiched between. Foams are 80 mm, and filter and gaskets are 81 mm in diameter. The 20 ppi foam is 20 mm deep and the 80 ppi foam is 12 mm deep. The filter is supported with a stainless-steel mesh of 81 mm diameter, adjacent to the final cone at the exit. The cone is further connected to a radial rotating union (not shown) and to the flow control of the apparatus. Air flow moves from left to right.

**Figure 3.**
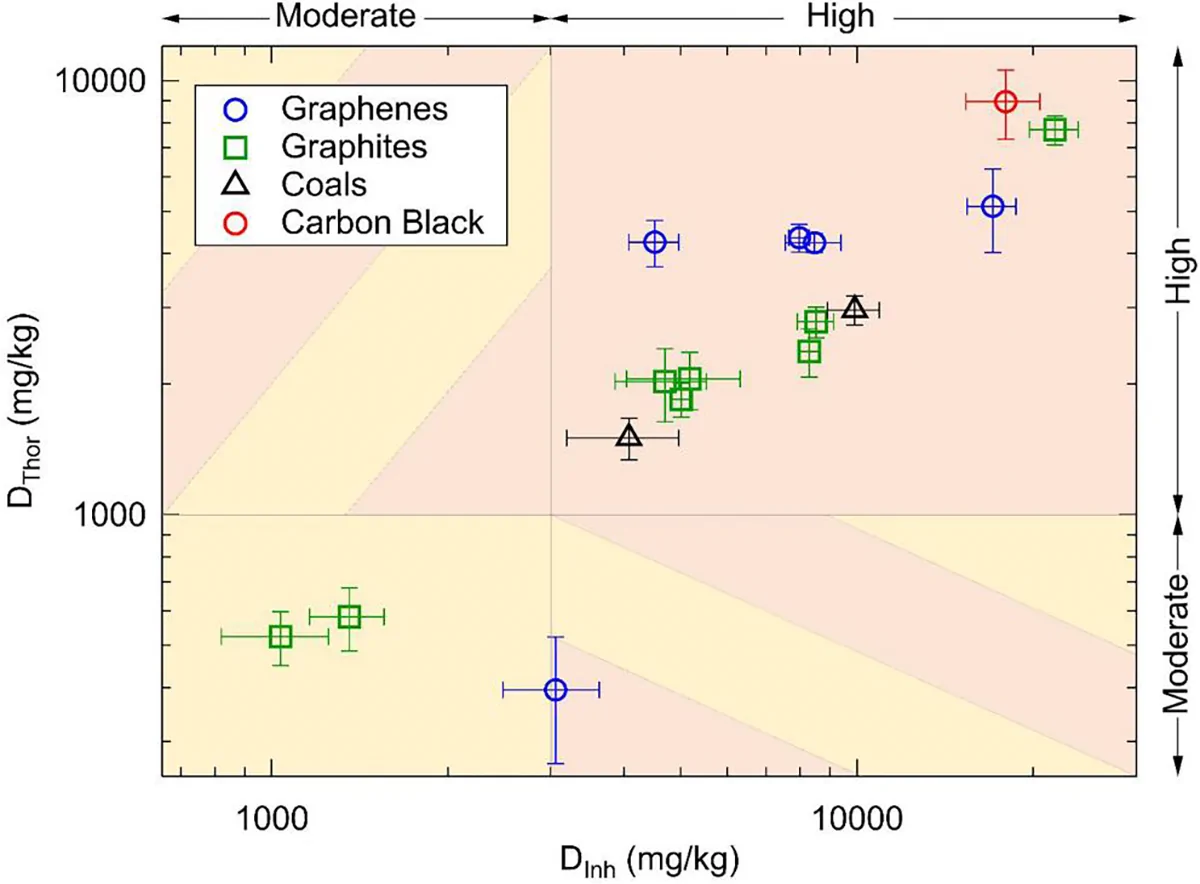
Relationship between thoracic (D_Thor_) and inhalable (D_Inh_) dustiness fractions for all sixteen carbonaceous test materials in this study. Test materials are grouped according to type: graphenes, graphites, coals, and carbon black. A logarithmic scale was utilized for both dustiness variables. Error bars represent ± one standard deviation from the mean. Dustiness classifications are illustrated by shaded regions and color scheme (Table 3).

**Figure 4.**
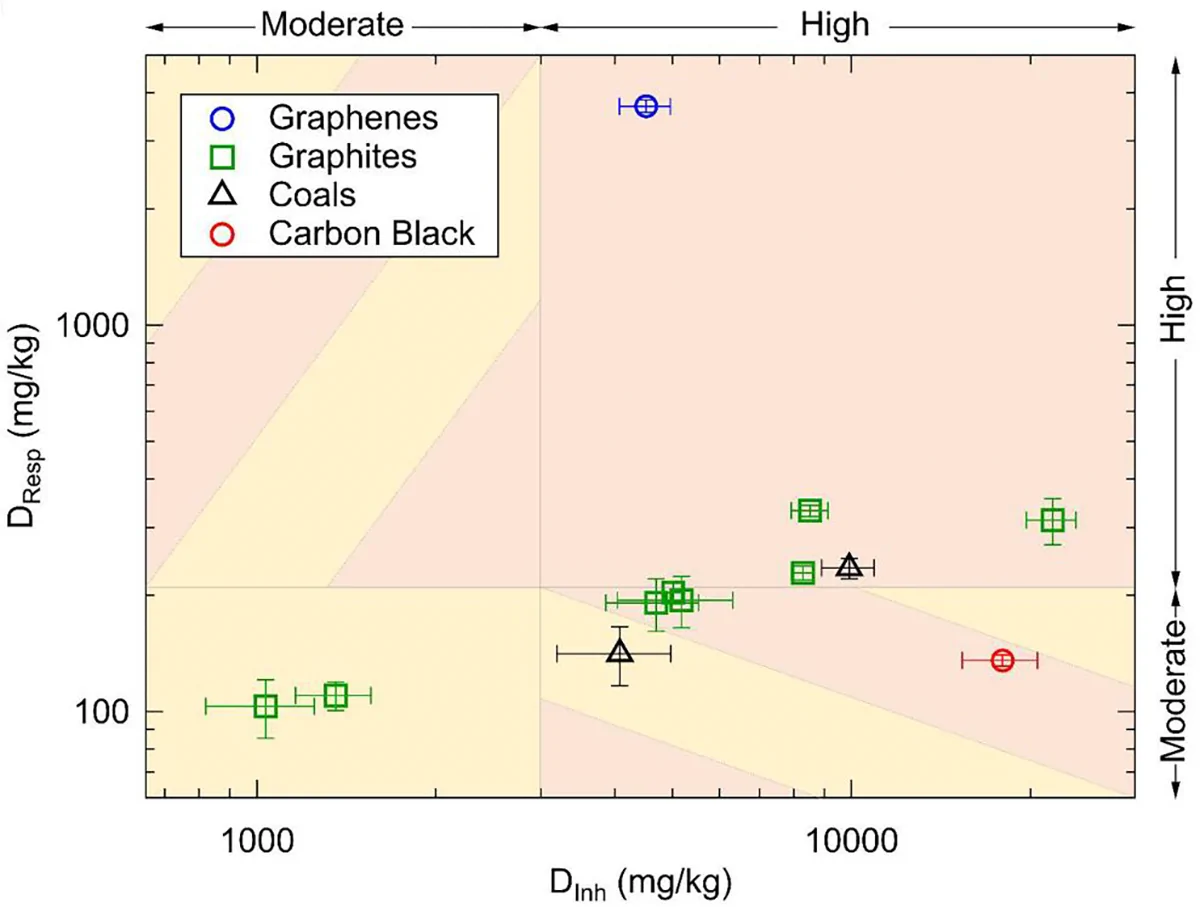
Relationship between respirable (D_Resp_) and inhalable (D_Inh_) dustiness for 12 test materials in this study. The B050 single layer graphene oxide is the only graphene material presented. Test materials are grouped according to material type: graphenes, graphites, coals, and carbon black. A logarithmic scale was utilized for both dustiness variables. Error bars represent ± one standard deviation from the mean. Dustiness classifications are illustrated by the shaded regions and color scheme (Table 3).

**Figure 5.**
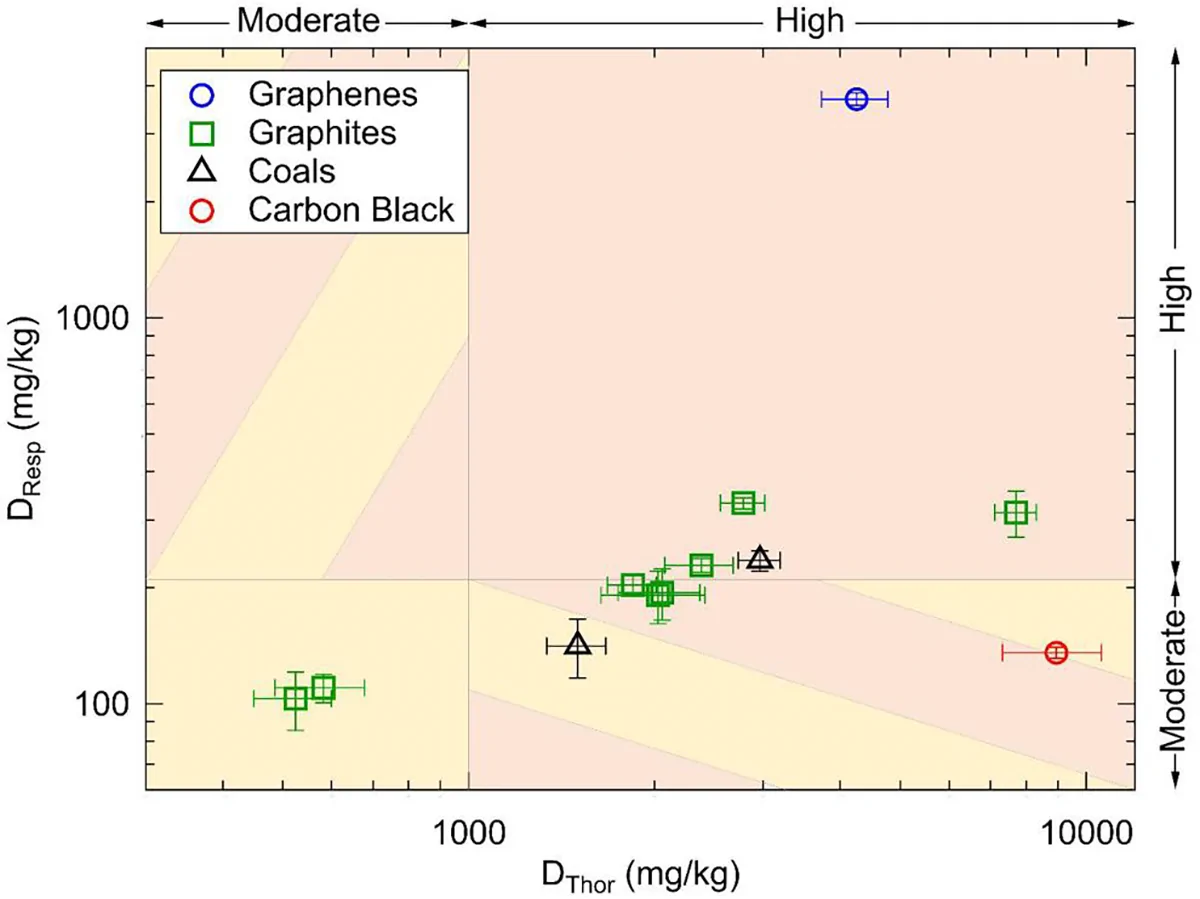
Relationship between respirable (D_Resp_) and thoracic (D_Thor_) dustiness fractions for 12 test materials in this study. The B050 single layer graphene oxide is the only graphene material presented. Test materials are grouped according to material type: graphenes, graphites, coals, and carbon black. A logarithmic scale was utilized for both dustiness variables. Error bars represent ± one standard deviation from the mean. Dustiness classifications are illustrated by the shaded regions and color scheme (Table 3).

**Figure 6.**
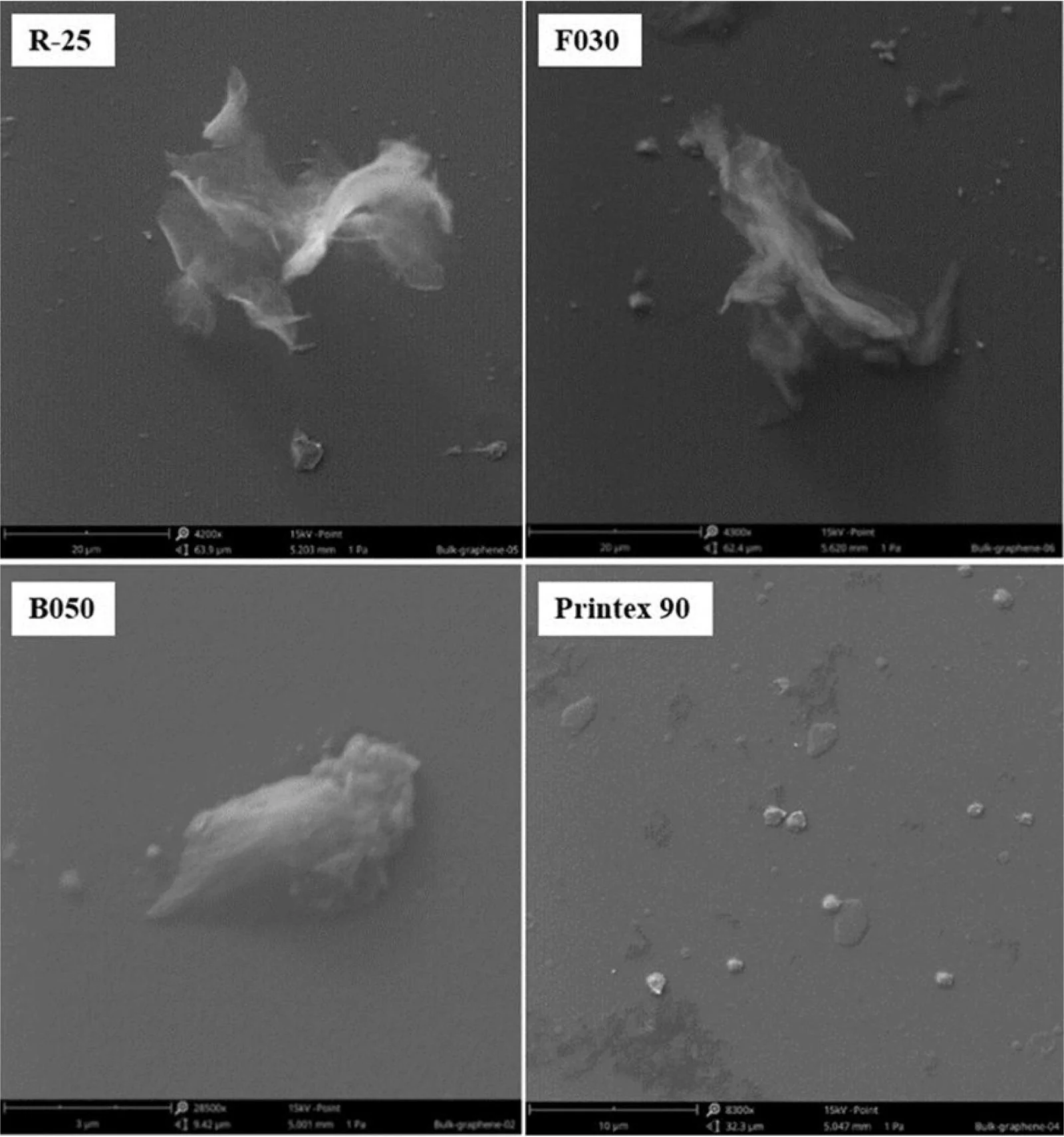
SEM micrographs from bulk nanoscale test materials. R-25 graphene nanoplatelets with a 20 μm scale bar, graphene oxide F030, with a 20 μm scale bar; single layer graphene oxide B050, with a 3 μm scale bar; and furnace carbon black Printex 90, with a 10 μm scale bar.

**Figure 7.**
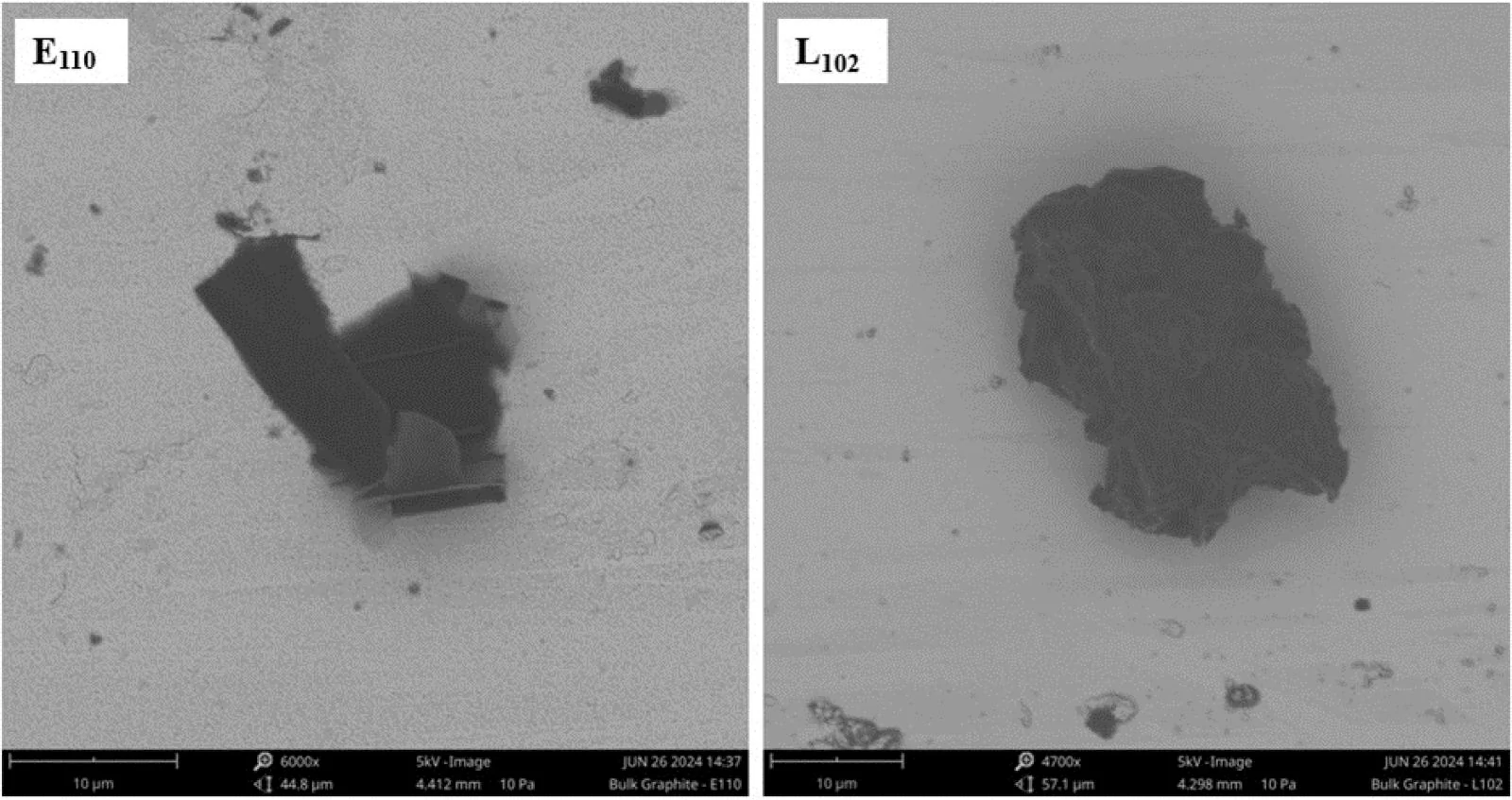
SEM micrographs from bulk graphite (microscale) test materials. E_110_ natural graphite and L_102_ synthetic graphite. Both micrographs possess a 10 μm scale bar.

**Table 1.** OELs for carbonaceous materials (NIOSH 2013, 2020; ACGIH 2026).

Carbonaceous Material	Size Fraction	OSHA PEL	MSHA PEL	NIOSH REL	ACGIH TLV

		mg/m^3^			
Graphite (synthetic)	Total/Inhalable	15			10
Graphite (synthetic)	Respirable	5			2
Graphite (natural)	Respirable	2.5		2.5	
Carbon Black	Total	3.5		3.5	
Coal Dust (bituminous or lignite)	Respirable		1.5*	0.9	0.9
Coal Dust (anthracite)	Respirable		1.5*		0.4
CNTs/CNFs	Respirable			0.001	

US Occupational Safety and Health Administration (OSHA), US Mine Safety and Health Administration (MSHA), US National Institute for Occupational Safety and Health (NIOSH) and the American Conference for Government Industrial Hygienists (ACGIH^®^). Permissible Exposure Limit (PEL), Recommended Exposure Limit (REL) and Threshold Limit Value (TLV^®^). Where multiple OELs are provided for a given material, a lower OEL is usually based on more recent risk assessments.

* Containing less than 5% crystalline silica.

**Table 2.** Sixteen carbonaceous test materials utilized in this study.

Test Material	Code	Manufacturing Approach

Graphene Nanoplatelets	R-25	Chemically exfoliated
	C-500	Mechanically exfoliated
Few-Layered Graphene	F030	Mechanically exfoliated
Single Layer Graphene Oxide	B050	Mechanically exfoliated
Edge-Oxidized Graphene	EOG	Mechanically exfoliated
Carbon Black	Printex 90	High temperature furnace
Natural Crystalline Flake Graphite	M_100_	Mined, milled, size classified
	F_117_	Mined, milled, size classified
	E_110_	Mined, milled, size classified
Synthetic Graphite	M_304_	High temperature, milled, size classified
	L_102_	High temperature, milled, size classified
	K_118_	High temperature, milled, size classified
	4,147	High temperature, milled, size classified
	240	High temperature, milled, size classified (240 mesh sieve)
Coal, Bag House Fines	BHF	Mined, pulverized, size classified (air)
Coal, PPC 1157	PPC 1157	Mined, pulverized

**Table 3.** Dustiness classification thresholds for the inhalable, thoracic, and respirable size fractions (EN15051–2, CEN 2013b).

EN15051-2 Dustiness Classification (mg/kg)			

	D_Inh_	D_Thor_	D_Resp_

Very Low	<300	<80	<10
Low	300–650	80–300	10–60
Moderate	>650–3,000	>300–1,000	>60–210
High	>3,000	>1,000	>210

**Table 4.** Carbonaceous test materials by type.

Test Material	Code		Dustiness			SSA	Moisture	Density
			mg/kg			m^2^/g	%	kg/m^3^
			D_Inh_	D_Thor_	D_Resp_			

Graphene								
Nanoplatelets	R-25	Mean	8,461	4,233	< LOD	35	0.11	26
		SD	926	208		1.0	0.016	0.65
	C-500	Mean	17,062	5,135	< LOD	595	4.9	152
		SD	1,645	1,114		2.7	0.021	2.8
Few-Layered	F030	Mean	7,975	4,349	< LOD	18	1.5	102
		SD	328	320		0.2	0.07	1.9
Oxides	B050	Mean	4,515	4,248	3,689	41	5.6	5.9
		SD	443	520	138	2.8	0.70	0.25
	EOG	Mean	3,056	395	< LOD	286	4.2	174
		SD	570	128		0.5	0.035	4.2
Carbon Black								
Furnace Black	Printex 90	Mean	17,955	8,947	136	273	2.5	135
		SD	2,598	1,625	4.4	2.5	0.013	4.0
Graphite								
Natural	M_100_	Mean	5,177	2,056	195	10	0.13	266
		SD	1,138	310	29	0.1	0.014	4.6
	F_117_	Mean	4,695	2,027	191	10	0.077	252
		SD	833	388	30	0.1	0.008	8.4
	E_110_	Mean	21,781	7,702	313	2.9	0.057	400
		SD	2,084	597	43	0.06	0.006	10
Synthetics	M_304_	Mean	1,035	524	103	26	0.19	279
		SD	215	75	18	0.3	0.005	4.8
	L_102_	Mean	5,013	1,844	204	11	0.014	374
		SD	215	167	13	0.07	0.006	12
	K_118_	Mean	1,358	582	110	2.0	0.063	676
		SD	198	96	9.4	0.1	0.003	7.9
	4,147	Mean	8,516	2,784	331	351	3.7	413
		SD	605	229	11	0.2	0.063	5.4
	240	Mean	8,281	2,380	229	19	0.15	504
		SD	355	301	10	0.02	0.034	10
Coal								
Bag House Fines	BHF	Mean	4,078	1,502	142	2.9	1.3	396
		SD	885	165	25	0.1	0.036	11
Pitts. Pulv. 1157	PPC 1157	Mean	9,911	2,963	236	0.9	1.3	592
		SD	1,004	231	14	0.1	0.014	9.1

Presented are dustiness values (mg/kg) for the inhalable (D_Inh_), thoracic (D_Thor_), and respirable (D_Resp_) size fractions, BET specific surface area, (SSA, m^2^/g), moisture content (%), and unconsolidated bulk density (kg/m^3^). Mean denotes the mean average, and SD is one standard deviation of the replicate measurements. Values are shaded corresponding to classification thresholds in Table 3.

## Data Availability

Data utilized in figures are available in tabulated format in this manuscript and supplementary materials.
