# Supplementary material for: Dustiness of graphene and graphite
Source: J Occup Environ Hyg. Author manuscript; Available in PMC 2026 Oct 14. (PMC13662783; doi:10.1080/15459624.2026.2692355)
Supplement: Supplementary file 1 [file NIHMS2210669-supplement-Supplemental.docx]

**Supplemental Materials**

Table S1: Test material provenance for the materials in this study.

| Test Material | Code | Manufacturer / Supplier |
| --- | --- | --- |
| Graphene Nanoplatelets | R-25 | XG Sciences Inc. |
|  | C-500 |  |
| Few-Layered Graphene | F030 | Global Graphene Group, Inc. |
| Single Layer Graphene Oxide | B050 |  |
| Edge-Oxidized Graphene | EOG | Garmor Inc. |
| Furnace Carbon Black | Printex 90 | Orion Engineered Carbons |
| Natural Crystalline Flake Graphite | M_100_ | Carbon Graphite Materials, Inc. |
|  | F_117_ |  |
|  | E_110_ |  |
| Synthetic Graphite | M_304_ | Carbon Graphite Materials, Inc. |
|  | L_102_ |  |
|  | K_118_ |  |
| Synthetic Graphite | 4147 | Asbury Carbons, Inc. |
| Synthetic Graphite, 240 mesh | 240 | James Durrans Group |
| Coal, Bag House Fines | BHF | Hadcel Coal |
| Coal, PPC 1157 | PPC 1157 |  |

*Graphene Nanoplatelets*. Grade R is available with average particle diameters of 7, 10, or 25 µm. R-25 refers to grade R, with an average particle diameter of 25 µm. Grade C is available with average specific surface areas of 300, 500, and 750 m²/g. C-500 refers to grade C, with an average specific surface area of 500 m^2^/g.

*Coals*. Test materials were supplied through the NIOSH Pittsburgh Mining Research Division and prepared by Hadcel Coal. Pittsburgh pulverized coal (PPC) 1157 is a bituminous rank and prepared by pulverizing mined lump coal. The bag house fines (BHF) sample was collected in the locale of the pulverizing operation from adjacent horizontal surfaces and is a float coal dust.

*Graphites*

Table S2: The following information was supplied by Carbon Graphite Materials Inc. for their graphite products.

| Test Material | Code | Density g/in^3^ | Approx. Size Distribution d_10_, d_50_, d_90_, (µm) |
| --- | --- | --- | --- |
| Natural Crystalline Flake Graphite | M_100_ | 2.5 | 2.5, 6.5, 14.5 |
|  | F_117_ | 3.0 | 3.0, 8.5, 18.0 |
|  | E_110_ | 6.0 | 24.4, 71, 132 |
| Synthetic Graphite | M_304_ | 2.15 | 1.3, 3.5, 8.8 |
|  | L_102_ | 3.5 | 3.5, 16.0, 43.5 |
|  | K_118_ | 10 | 55, 97, 162 |

Table S3: Gravimetric mass and bulk unconsolidated density for test materials initially loaded into the test apparatus and subsequently collected on media. 20 and 80 ppi refer to the porosities of the particle size-selective nickel foams. (* mean values that fall below the limit of detection.)

| Test Material |  | Gravimetric Masses | | | | Bulk Density |
| --- | --- | --- | --- | --- | --- | --- |
|  |  | Loaded | 20 ppi | 80 ppi | Filter |  |
|  |  | g | mg | mg | µg | kg/m^3^ |
| Graphene |  |  |  |  |  |  |
| R-25 | Mean | 0.92 | 3.9 | 3.9 | *0.03 | 26 |
|  | Std. Dev. | 0.02 | 0.71 | 0.19 | 0.00 | 0.65 |
| C-500 | Mean | 5.3 | 63 | 27 | *0.14 | 152 |
|  | Std. Dev. | 0.1 | 11 | 6.1 | 0.04 | 2.8 |
| F030 | Mean | 3.6 | 13 | 16 | *0.54 | 102 |
|  | Std. Dev. | 0.1 | 2.1 | 1.1 | 0.10 | 1.9 |
| B050 | Mean | 0.21 | 0.11 | 0.11 | 0.76 | 5.9 |
|  | Std. Dev. | 0.01 | 0.05 | 0.10 | 0.01 | 0.25 |
| EOG | Mean | 6.1 | 16 | 2.4 | *0.22 | 174 |
|  | Std. Dev. | 0.1 | 3.1 | 0.8 | 0.01 | 4.2 |
| Carbon Black |  |  |  |  |  |  |
| Printex 90 | Mean | 4.7 | 42 | 42 | 0.64 | 135 |
|  | Std. Dev. | 0.1 | 7.4 | 7.2 | 0.01 | 4.0 |
| Natural Graphite |  |  |  |  |  |  |
| M_100_ | Mean | 9.3 | 29 | 17 | 1.81 | 266 |
|  | Std. Dev. | 0.2 | 8.2 | 2.6 | 0.28 | 4.6 |
| F_117_ | Mean | 8.8 | 23 | 16 | 1.68 | 252 |
|  | Std. Dev. | 0.3 | 3.9 | 2.9 | 0.23 | 8.4 |
| E_110_ | Mean | 14.0 | 209 | 104 | 4.40 | 400 |
|  | Std. Dev. | 0 | 30 | 10 | 0.68 | 10 |
| Synthetic Graphite |  |  |  |  |  |  |
| M_304_ | Mean | 9.8 | 5.0 | 4.1 | 1.01 | 279 |
|  | Std. Dev. | 0.2 | 1.4 | 0.6 | 0.18 | 4.8 |
| L_102_ | Mean | 13.1 | 41 | 21 | 2.67 | 374 |
|  | Std. Dev. | 0.4 | 2.9 | 2.0 | 0.21 | 12 |
| K_118_ | Mean | 24 | 18 | 11 | 2.61 | 676 |
|  | Std. Dev. | 0.3 | 3.4 | 2.2 | 0.22 | 7.9 |
| 4147 | Mean | 14.4 | 83 | 35 | 4.78 | 413 |
|  | Std. Dev. | 0.2 | 5.0 | 2.7 | 0.14 | 5.4 |
| 240 | Mean | 17.7 | 104 | 38 | 4.04 | 504 |
|  | Std. Dev. | 0.36 | 10 | 5.2 | 0.15 | 10 |
| Coal |  |  |  |  |  |  |
| BHF | Mean | 13.9 | 36 | 19 | 1.96 | 396 |
|  | Std. Dev. | 0.4 | 12 | 2.0 | 0.33 | 11 |
| PPC 1157 | Mean | 21 | 144 | 57 | 4.88 | 592 |
|  | Std. Dev. | 0.3 | 17 | 5.1 | 0.28 | 9.1 |
| Detection Limits | - | 1.4E-06 | 0.097 | 0.085 | 0.57 | 4.0E-05 |

Test material masses loaded in the apparatus ranged from 0.21 to 24 g. Masses collected on the 20ppi foams ranged from 0.112 to 209 mg. Masses collected on the 80ppi foams ranged from 0.114 to 104 mg. Masses collected on the glass microfiber filters were all under 5.0 µg and included values below our 0.57 µg gravimetric limit of detection (see Figure S1).

Figure S1: Scatter plot of respirable mass collected on filters (µg) versus the bulk unconsolidated density of the test materials loaded into the test apparatus (kg/m^3^). Data from Table S3. EN15051-2 stipulates a fixed 35 mL test material loading volume. The shaded area bounded by 200 kg/m^3^, 1.0 µg and the axes, denotes an approximate area that is more challenging to quantify gravimetrically. It encompasses all nanoscale test materials (graphenes and carbon black) in this study.
